# Monocytes and parturition: Linking prolonged labor to immune dysregulation

**DOI:** 10.1097/MD.0000000000042351

**Published:** 2025-04-25

**Authors:** Emmanuel Ifeanyi Obeagu

**Affiliations:** a Department of Biomedical Laboratory Science, Africa University, Mutare, Zimbabwe.

**Keywords:** cytokines, immune dysregulation, monocytes, parturition, prolonged labor

## Abstract

Monocytes play a pivotal role in the immune regulation of labor, contributing to processes like cervical ripening, uterine contractility, and the initiation of parturition. During labor, monocytes are recruited to the cervix and uterus, where they undergo activation and release pro-inflammatory cytokines that help mediate uterine contractions and facilitate cervical remodeling. However, immune dysregulation involving excessive or insufficient monocyte activation can contribute to complications such as prolonged labor. The imbalance in cytokine production and monocyte dysfunction is thought to be a key factor in delayed labor, making monocytes critical targets for understanding and managing labor abnormalities. Recent studies have explored the potential of monocyte-related biomarkers as predictive tools for identifying women at risk for prolonged labor. Monocyte subsets and cytokine profiles, including markers such as CD14, CD16, interleukin-1β, tumor necrosis factor-alpha, and interleukin-6, offer valuable insights into the inflammatory status of the cervix and uterus. The ability to assess monocyte function and cytokine levels may provide early indications of immune dysregulation, allowing for timely interventions to prevent prolonged labor and reduce complications. Advances in diagnostic technologies, such as cytokine assays and flow cytometry, are improving our ability to monitor monocyte activity during labor in real time.

## 1. Introduction

Labor is a multifaceted physiological process that involves coordinated hormonal, mechanical, and immune factors. It marks the transition from pregnancy to the birth of the fetus and is essential for the survival of both the mother and the neonate. The process of parturition is initiated by complex signaling pathways, including those that mediate inflammation and immune responses. Monocytes, as key components of the innate immune system, play a significant role in the inflammatory processes that drive labor. These cells are recruited to the cervix and uterus, where they become activated and produce a range of cytokines and other immune mediators that contribute to uterine contractions, cervical ripening, and tissue remodeling.^[1,2]^ Monocytes are versatile cells that can differentiate into macrophages and dendritic cells, which are involved in both pro-inflammatory and anti-inflammatory responses. During labor, they help mediate the local immune response by releasing cytokines such as interleukin-6 (IL-6), tumor necrosis factor-alpha (TNF-α), and interleukin-1β (IL-1β). These cytokines promote inflammation, which is necessary for cervical remodeling and the initiation of uterine contractions. However, when this immune response is dysregulated, it can result in complications such as prolonged labor, a condition in which the normal progression of labor is delayed beyond a typical timeframe.^[3,4]^ Prolonged labor is associated with significant risks for both the mother and the fetus, including maternal exhaustion, infection, hemorrhage, and fetal distress. Immune dysregulation, particularly involving monocytes, has been implicated in the pathophysiology of prolonged labor. When monocyte recruitment and activation become imbalanced, it can lead to either excessive inflammation or inadequate immune response, both of which can hinder labor progression. This review aims to explore the role of monocytes in parturition and their involvement in the immune dysregulation that may contribute to prolonged labor. By understanding these mechanisms, we can identify potential biomarkers for early detection and intervention, ultimately improving maternal and neonatal outcomes.^[5,6]^

The immune system plays an essential role in the initiation and progression of labor. In normal labor, the balance of pro-inflammatory and anti-inflammatory responses is tightly regulated to ensure a coordinated process. Monocytes are recruited to the cervix and uterus, where they undergo differentiation and activation in response to various signals. Once activated, they release cytokines and other mediators that help remodel the cervix and trigger uterine contractions. This immune activation is necessary for the successful completion of labor, but when it becomes dysregulated, it can lead to complications such as prolonged labor.^[7,8]^ Monocytes are typically activated in response to signals from the fetus and placenta, which trigger the release of various cytokines and other mediators. These signals include changes in the levels of pro-inflammatory cytokines, mechanical stretch of the uterus, and the release of hormones like oxytocin. Once activated, monocytes produce cytokines that act on the cervix and uterus to facilitate remodeling and contractions. For example, TNF-α, IL-1β, and IL-6 are crucial for the activation of matrix metalloproteinases (MMPs), which help degrade the extracellular matrix and allow for cervical softening and uterine contraction.^[9,10]^ However, when the immune system’s regulation is disturbed, monocyte function may be altered, leading to pathological changes that affect labor progression. In prolonged labor, the immune response may be either exaggerated or insufficient, disrupting the delicate balance necessary for labor to proceed normally. Elevated levels of pro-inflammatory cytokines or a lack of resolution of inflammation can contribute to uterine dysfunction or cervical failure, both of which can result in delayed labor.^[11]^ In cases of prolonged labor, the recruitment and activation of monocytes can be persistently elevated, resulting in excessive cytokine production. This chronic inflammation can lead to uterine muscle fatigue, reducing the efficiency of contractions and delaying the progression of labor. On the other hand, insufficient monocyte activation may result in a lack of inflammatory signals that are necessary for cervical ripening and uterine contractility. Either scenario can contribute to prolonged labor, underscoring the importance of proper monocyte function in the normal progression of parturition.^[12,13]^

Monocytes are not only involved in producing cytokines but also in other processes crucial for labor. These cells contribute to tissue remodeling through the production of enzymes like MMPs, which degrade the extracellular matrix in the cervix and uterus, facilitating cervical dilation and uterine contractility. Inadequate monocyte-derived MMP activity can lead to a rigid cervix and poor uterine contractions, both of which are associated with prolonged labor. The ability of monocytes to modulate tissue remodeling is therefore a key component of their role in parturition.^[14,15]^ The role of monocytes in labor has led to increasing interest in their potential as biomarkers for predicting and diagnosing labor complications. By analyzing the activation status of monocytes and the cytokine profiles they produce, clinicians could gain valuable insights into the inflammatory state of the cervix and uterus. This could allow for the early identification of women at risk for prolonged labor, enabling more targeted and timely interventions. Biomarkers such as CD14, CD16, IL-1β, TNF-α, and IL-6 have been investigated for their potential to predict labor outcomes, and advances in diagnostic techniques like flow cytometry and cytokine assays are improving our ability to monitor monocyte activity during labor.^[16,17]^ The identification of reliable biomarkers for prolonged labor is essential for improving clinical management. By profiling the immune response and monocyte activation, clinicians could better predict labor progression and intervene when necessary. For instance, therapies that modulate monocyte function, either by promoting or inhibiting cytokine production, could help restore immune balance and promote labor progression. The development of such therapies holds promise for improving outcomes in women with prolonged labor, potentially reducing the need for more invasive interventions such as cesarean delivery.^[18,19]^

### 1.1. Aim

The aim of this review is to explore the role of monocytes in the pathophysiology of prolonged labor, focusing on their recruitment, activation, and the cytokine-mediated processes that contribute to immune dysregulation.

### 1.2. Rationale of the review

Prolonged labor is a common and complex obstetric complication that can lead to adverse maternal and neonatal outcomes, including increased rates of cesarean sections, birth injuries, and postpartum complications. The immune system, particularly monocytes, plays a crucial role in the regulation of labor, influencing key processes such as cervical ripening, uterine contractions, and the resolution of inflammation necessary for successful parturition. However, the mechanisms through which monocytes contribute to labor progression, particularly in cases of prolonged labor, remain poorly understood.^[20]^ Monocyte dysregulation, characterized by altered recruitment, activation, and cytokine production, has been implicated in various inflammatory disorders, including those affecting pregnancy. Recent studies suggest that immune system imbalances, particularly involving monocytes and their cytokine signaling, could be a significant factor in the pathophysiology of prolonged labor. These observations highlight the need for a comprehensive understanding of the role of monocytes in this condition, which could lead to the identification of novel biomarkers and therapeutic targets.^[21]^ This review aims to provide a mechanistic insight into how monocytes and their immune responses contribute to prolonged labor. By synthesizing current knowledge, we seek to enhance the understanding of the immunological factors that drive labor complications and explore how monocytes might serve as biomarkers to predict and manage prolonged labor more effectively. Furthermore, the review will identify knowledge gaps and offer recommendations for future research, ultimately contributing to better clinical outcomes for pregnant women.

## 2. Review methodology

This narrative review was conducted to synthesize and critically evaluate existing literature on the role of monocytes in prolonged labor, with a particular focus on their involvement in immune dysregulation and cytokine production. The review methodology followed a systematic approach to ensure comprehensive coverage of relevant studies while maintaining clarity and precision in the synthesis of findings.

### 2.1. Literature search strategy

A comprehensive literature search was conducted using multiple databases, including PubMed, Scopus, Google Scholar, and Web of Science. The search was limited to peer-reviewed articles published in English from the past 15 years to ensure the inclusion of the most relevant and up-to-date studies. Keywords and search terms included combinations of “monocytes,” “prolonged labor,” “immune dysregulation,” “cytokine storm,” “cervical ripening,” “uterine contractility,” and “parturition,” among others. This search aimed to identify studies that investigated the role of monocytes in labor, their cytokine profiles, and their contributions to complications such as prolonged labor.

### 2.2. Inclusion and exclusion criteria

The inclusion criteria were as follows:

1. Studies examining the role of monocytes in pregnancy, labor, and delivery.
2. Articles focusing on the immune dysregulation observed in prolonged labor.
3. Human studies, animal studies, and in vitro studies were included.
4. Clinical trials, observational studies, and reviews related to cytokine imbalances in labor.

The exclusion criteria included:

1. Studies that did not directly address monocytes or immune cells in the context of labor.
2. Studies focusing solely on non-immune-related causes of prolonged labor.
3. Articles that were not available in full text or published in non-peer-reviewed sources.

## 3. Monocyte recruitment and activation during labor

Monocytes are key players in the inflammatory processes that initiate and regulate labor. The recruitment and activation of monocytes during labor are essential for cervical ripening, uterine contractility, and the inflammatory responses required for a successful delivery. These immune cells, which circulate in the bloodstream, are rapidly recruited to the cervix and uterus in response to signals from the fetus, placenta, and uterine tissue. Upon reaching the site of labor, monocytes undergo differentiation into macrophages and dendritic cells, which are capable of producing a wide array of pro-inflammatory cytokines, enzymes, and other molecules that contribute to the labor process.^[22]^ The recruitment of monocytes during labor is primarily driven by chemokines and cytokines released from the cervix, uterus, and fetal membranes. One of the primary signals for monocyte recruitment is the release of pro-inflammatory cytokines, such as IL-1β, TNF-α, and IL-6, which are produced in response to uterine contractions and cervical remodeling. These cytokines activate local immune responses and induce the release of chemokines like monocyte chemoattractant protein-1, which specifically attract monocytes to the cervix and uterus. This process is essential for initiating the inflammatory cascade that softens and dilates the cervix, as well as for preparing the uterine muscle for contractions.^[23]^ Once monocytes arrive at the cervix and uterus, they undergo activation, which enhances their ability to produce pro-inflammatory cytokines, MMPs, and other factors that promote uterine contractility and cervical ripening. For example, monocytes produce MMPs, which break down the extracellular matrix of the cervix, allowing it to soften and dilate in preparation for delivery. Additionally, activated monocytes release cytokines such as IL-6, IL-8, and TNF-α, which promote uterine contractions and coordinate the inflammatory responses necessary for the expulsion of the fetus. This activation process is crucial for the timely progression of labor and the eventual delivery of the neonate.^[24]^

Monocyte activation is also tightly regulated to ensure that labor progresses in a controlled manner. The inflammatory response needs to be balanced, as excessive or insufficient immune activation can lead to complications, such as prolonged labor. If monocytes are overly activated, excessive cytokine production may result in exaggerated uterine contractions or uterine hyperstimulation, which can cause fetal distress or maternal injury. Conversely, inadequate monocyte activation can result in insufficient cervical ripening or weak contractions, leading to stalled labor or failure to progress. Therefore, the regulation of monocyte recruitment and activation is critical for ensuring that labor proceeds efficiently and safely.^[25]^ The recruitment and activation of monocytes are influenced by a range of signaling molecules and immune modulators. For example, prostaglandins, which are released in response to hormonal signals like oxytocin, can enhance monocyte activation. Additionally, interactions between monocytes and other immune cells, such as neutrophils and T-cells, help to coordinate the immune response during labor. The dynamic interaction between monocytes and these cells is crucial for maintaining the balance between pro-inflammatory and anti-inflammatory signals, which ultimately determines the success of labor progression.^[25]^ As labor progresses, monocytes play a key role in regulating uterine tone and cervical dilatation. They achieve this by producing cytokines and enzymes that modulate uterine smooth muscle contractility. For instance, TNF-α and IL-1β have been shown to increase uterine contractions by stimulating prostaglandin release, which in turn activates the smooth muscle cells of the uterus. In addition to promoting contractions, the cytokines released by monocytes also induce the release of other factors, such as nitric oxide and relaxin, which help to relax the cervix and prepare the uterus for the expulsive phase of labor.^[26]^ Moreover, the recruitment of monocytes is influenced by the fetal membranes, which release pro-inflammatory mediators in response to mechanical stretch and other signals during labor. As the fetus moves within the uterus, mechanical forces are exerted on the cervix and uterine tissues, triggering the release of signaling molecules like cytokines, which then attract monocytes to the area. This process not only facilitates labor but also prepares the body for postpartum recovery and immune defense.^[27]^

## 4. Cytokine imbalance and immune dysregulation in prolonged labor

Prolonged labor is a complex obstetric complication that can have significant maternal and neonatal consequences. At the heart of labor progression lies a finely tuned immune response, where cytokines and immune cells orchestrate the inflammatory processes necessary for cervical ripening, uterine contractions, and eventual delivery. In a typical labor scenario, cytokines such as IL-1β, TNF-α, and IL-6 are released to initiate labor, while other cytokines like IL-10 and transforming growth factor-beta help resolve inflammation and restore homeostasis postdelivery. However, in cases of prolonged labor, this immune balance is often disrupted, leading to cytokine imbalances that contribute to a failure in labor progression.^[28]^ Cytokine imbalance can manifest as either excessive or insufficient inflammatory responses. In some cases, a hyper-inflammatory state characterized by excessive cytokine production, particularly pro-inflammatory mediators like IL-6 and TNF-α, may result in excessive uterine contractions, leading to fetal distress or maternal complications. This exaggerated response can be triggered by various factors, including infections, placental abnormalities, or uterine overstretch. The resulting overactivation of immune pathways not only impedes normal cervical remodeling and uterine contractility but can also cause maternal systemic inflammation, increasing the risk of complications such as chorioamnionitis and sepsis.^[29]^ Conversely, an insufficient inflammatory response may also hinder labor progression. A deficiency in key pro-inflammatory cytokines can lead to inadequate uterine contractility, cervical ripening, and insufficient effacement, contributing to prolonged labor. Inflammatory mediators such as IL-1β and prostaglandins play an essential role in stimulating uterine contractions and cervical softening. When these cytokines are underproduced or improperly regulated, the labor process may fail to progress at a normal pace, resulting in delayed or arrested labor. This lack of immune response can also prevent the adequate activation of MMPs, which are essential for cervical remodeling, further impeding the labor process.^[30]^

Monocytes are integral in mediating these cytokine responses during labor. Upon activation, monocytes release a variety of cytokines that influence uterine contractility and cervical ripening. However, the dysregulation of monocyte function in prolonged labor contributes significantly to immune imbalance. Studies have shown that in cases of prolonged labor, monocytes exhibit altered cytokine profiles, producing higher levels of pro-inflammatory cytokines while failing to adequately release anti-inflammatory mediators. This dysregulated cytokine release may prolong labor by exacerbating inflammation in the uterine environment, inhibiting the normal progression of labor. For instance, an overproduction of IL-6 and TNF-α can induce an excessive inflammatory response, potentially leading to uterine hyperactivity and impaired cervical ripening.^[31]^ In addition to cytokine dysregulation, prolonged labor is also associated with impaired resolution of inflammation. In normal labor, the inflammatory response is tightly regulated, with anti-inflammatory cytokines like IL-10 and transforming growth factor-beta playing a critical role in damping down excessive inflammation once delivery is imminent. However, in prolonged labor, the balance between pro- and anti-inflammatory cytokines is skewed, preventing proper resolution of inflammation. This persistent, unregulated immune response can hinder uterine contractions and cervical effacement, preventing the normal progression of labor.^[32]^ Cytokine imbalance and immune dysregulation in prolonged labor are also influenced by systemic factors such as maternal infection, stress, and nutritional status. Infections, particularly chorioamnionitis and bacterial vaginosis, are common contributors to immune dysregulation in labor. These infections can trigger an exaggerated inflammatory response, leading to cytokine storms that disrupt normal labor mechanisms. Additionally, maternal stress and nutritional deficiencies have been shown to alter immune function, which can also contribute to cytokine imbalances and labor abnormalities. For example, maternal stress can elevate cortisol levels, which may suppress immune function and impair the ability to resolve inflammation in the uterine environment.^[33]^ The role of the placental and fetal signals in cytokine dysregulation also cannot be overlooked. The placenta releases a variety of signaling molecules that influence both maternal and fetal immune responses. In cases of placental insufficiency or other placental abnormalities, the cytokine signaling between the fetus and mother may become imbalanced, leading to prolonged labor. Placental production of cytokines such as IL-6 and IL-8 can contribute to both local and systemic inflammation, disrupting the delicate balance of immune regulation required for the timely progression of labor.^[34]^

## 5. Monocytes as predictive biomarkers for prolonged labor

Prolonged labor is a significant obstetric complication that can have adverse effects on both maternal and neonatal health. The pathophysiology of prolonged labor is complex, involving multiple physiological and immunological processes. One of the key players in these processes is the monocyte, a type of white blood cell that plays a central role in immune regulation and inflammatory responses. Recent studies suggest that monocytes, through their recruitment, activation, and cytokine production, may serve as predictive biomarkers for prolonged labor, offering potential diagnostic and therapeutic insights.^[35]^ Monocytes are crucial for initiating and regulating the immune response during labor. They are recruited to the cervix and uterus during labor, where they participate in inflammation, tissue remodeling, and uterine contractions. These immune cells respond to signaling molecules such as cytokines, chemokines, and hormones, which play a critical role in cervical ripening and the initiation of labor. In normal labor, monocytes contribute to the inflammation required for uterine contraction and cervical softening, which facilitates the progression of labor. However, in cases of prolonged labor, this process is disrupted, and monocytes may become dysregulated, leading to altered cytokine profiles that affect labor progression.^[36]^ Research indicates that monocytes in women with prolonged labor may exhibit different functional profiles compared with those in women experiencing normal labor. Specifically, the activation and cytokine production of monocytes can become imbalanced, leading to either an exaggerated or insufficient inflammatory response. In cases of excessive inflammation, monocytes may overproduce pro-inflammatory cytokines such as IL-6, TNF-α, and IL-1β, which can contribute to uterine hyperactivity, cervical dystocia, and other complications. Conversely, insufficient activation of monocytes may result in inadequate inflammation, hindering uterine contractions and cervical remodeling. Monitoring the functional status of monocytes, including their cytokine production patterns, could potentially serve as an early indicator of labor complications.^[37–40]^

Monocytes may also be involved in the regulation of immune tolerance during pregnancy. During normal pregnancy, there is a delicate balance between immune activation and suppression, allowing the body to tolerate the fetus while defending against pathogens. However, in prolonged labor, this balance is often disrupted, with monocytes playing a central role in the failure to resolve inflammation. The imbalance of immune responses, particularly the failure of monocytes to produce sufficient anti-inflammatory cytokines such as IL-10, could result in a chronic inflammatory environment that impedes labor progression. By assessing the cytokine profiles of monocytes, it may be possible to predict the risk of prolonged labor and identify women who may benefit from early interventions.^[4,41,42]^ Another critical aspect of monocyte involvement in prolonged labor is their role in cervical remodeling and uterine contractility. Monocytes influence the extracellular matrix and the release of MMPs, enzymes necessary for cervical softening and uterine contractions. Dysregulation of this process, such as an imbalance in the secretion of MMPs, could result in cervical rigidity and a failure to progress in labor. Monitoring monocyte activity and MMP levels could, therefore, provide valuable predictive information regarding the likelihood of prolonged labor.^[43–46]^ Emerging research suggests that specific monocyte-derived biomarkers, such as cytokines, chemokines, and cell surface markers, may be useful in predicting labor outcomes. By identifying biomarkers associated with monocyte activation and function, it may be possible to develop diagnostic tools to assess the risk of prolonged labor. For example, higher levels of pro-inflammatory cytokines, such as IL-6 and TNF-α, or the presence of specific monocyte subtypes may correlate with an increased risk of prolonged labor. Similarly, alterations in the monocyte-to-lymphocyte ratio, a marker of systemic inflammation, have been proposed as a potential predictive biomarker for adverse pregnancy outcomes, including prolonged labor.^[47–49]^

In addition to cytokines and cell surface markers, recent studies have explored the potential of epigenetic modifications in monocytes as predictive biomarkers for prolonged labor. Epigenetic changes, such as DNA methylation and histone modifications, can influence gene expression in monocytes and alter their functional responses. These modifications may be influenced by maternal factors such as stress, nutrition, and infections, which can, in turn, impact the immune response during labor. By studying epigenetic changes in monocytes, it may be possible to identify women at risk for prolonged labor long before clinical symptoms manifest.^[50]^ The use of monocytes as predictive biomarkers for prolonged labor has significant clinical implications. Early identification of women at risk for prolonged labor could enable timely interventions, such as the administration of immunomodulatory therapies or the use of medications that promote uterine contractions. For example, if a woman is found to have elevated levels of pro-inflammatory cytokines or altered monocyte profiles, clinicians may consider more intensive monitoring or the use of labor augmentation strategies. Moreover, personalized treatment approaches based on monocyte-derived biomarkers could reduce the need for unnecessary interventions, such as cesarean sections, and improve maternal and neonatal outcomes.^[51,52]^

## 6. Recommendations

### 6.1. Expansion of research into monocyte subtypes and function

Future studies should focus on further characterizing the different subtypes of monocytes and their specific roles in the pathophysiology of prolonged labor. Detailed investigations into the functional profiles of these subtypes could help identify biomarkers that are more specific and reliable in predicting labor progression. Understanding how different monocyte subtypes contribute to inflammation, cervical remodeling, and uterine contractility could refine diagnostic approaches.

### 6.2. Development of monocyte-based diagnostic tools

The development of simple and cost-effective diagnostic tools for monitoring monocyte-derived biomarkers, such as cytokines, chemokines, and cell surface markers, should be prioritized. These tools could aid clinicians in identifying women at risk for prolonged labor early in pregnancy or during the early stages of labor. Integrating monocyte-based diagnostics with existing labor monitoring systems may improve the overall clinical management of pregnant women.

### 6.3. Longitudinal studies to understand cytokine dynamics

Longitudinal studies should be conducted to monitor cytokine profiles and monocyte activity throughout the: course of pregnancy and labor. This would allow for a more comprehensive understanding of how cytokine imbalances evolve and their relationship with labor duration. By tracking these markers over time, researchers can better predict labor outcomes and identify early indicators of complications.

### 6.4. Integration of monocyte-based biomarkers with clinical factors

Monocyte-based biomarkers should be considered as part of a broader diagnostic framework, incorporating other clinical factors such as maternal health, fetal well-being, and previous obstetric history. Integrating these biomarkers into a multi-factorial predictive model could enhance clinical decision-making and guide personalized treatment strategies for women at risk of prolonged labor.

### 6.5. Exploration of monocyte epigenetics in labor

Further investigation into the role of epigenetic modifications in monocytes, such as DNA methylation and histone modifications, may uncover new biomarkers linked to prolonged labor. Research into how environmental factors, such as maternal stress, infection, or nutrition, influence monocyte epigenetics could provide new insights into the mechanisms behind labor progression and lead to novel therapeutic interventions.

### 6.6. Clinical trials of immunomodulatory therapies

Clinical trials are needed to evaluate the effectiveness of immunomodulatory therapies that target monocyte activity and cytokine production. Interventions that aim to balance the inflammatory response may help to optimize labor progression and reduce the incidence of prolonged labor. Such therapies could be developed to either enhance or suppress specific aspects of monocyte function, depending on the identified dysregulation.

### 6.7. Increased collaboration between obstetrics and immunology

A multidisciplinary approach, involving collaboration between obstetricians, immunologists, and researchers, is essential for advancing our understanding of the role of monocytes in prolonged labor. This collaboration can help bridge the gap between immunological research and clinical practice, facilitating the development of more effective diagnostic and therapeutic interventions.

### 6.8. Personalized approaches to labor management

Based on findings from monocyte-based biomarkers, personalized strategies for labor management should be developed. These strategies could be tailored to individual risk profiles, taking into account both maternal and immune factors. Personalized care would not only improve outcomes for women with prolonged labor but also minimize unnecessary interventions, such as cesarean sections, when possible.

### 6.9. Public health and education initiatives

Raising awareness among healthcare providers about the potential role of monocytes in labor progression and complications is important. Educational programs could help ensure that obstetricians are aware of emerging research in immunology and how it may inform their practice. This could also extend to educating pregnant women, particularly those with risk factors for prolonged labor, on the importance of monitoring immune health throughout pregnancy.

### 6.10. Integration of monocyte biomarkers into routine obstetric care

Lastly, the incorporation of monocyte-based biomarkers into routine obstetric care should be explored, particularly for women who are at risk of prolonged labor due to preexisting health conditions. This could involve incorporating simple blood tests to assess monocyte function and cytokine levels at various points during pregnancy and labor. Such measures may not only predict labor complications but also guide early interventions, improving maternal and neonatal outcomes.

## 7. Conclusion

Monocytes play a pivotal role in the immune regulation of labor, and their dysregulation is increasingly recognized as a contributing factor to prolonged labor. Through their involvement in the production of cytokines and chemokines, monocytes influence crucial processes such as cervical ripening, uterine contractility, and the balance of inflammation necessary for normal labor progression. The alteration in monocyte function—whether through excessive pro-inflammatory responses or insufficient immune activation—can lead to disruptions in these processes, ultimately resulting in prolonged labor and its associated complications. Emerging evidence suggests that monitoring monocyte activity and cytokine profiles may provide valuable predictive biomarkers for identifying women at risk of prolonged labor. This approach offers the potential for earlier diagnosis and more personalized management strategies, which could improve maternal and neonatal outcomes. Moreover, further research into the role of epigenetic modifications in monocytes and their impact on labor could unveil novel biomarkers and therapeutic targets, advancing our understanding of the immunological underpinnings of labor complications.

## Author contributions

**Conceptualization:** Emmanuel Ifeanyi Obeagu.

**Methodology:** Emmanuel Ifeanyi Obeagu.

**Resources:** Emmanuel Ifeanyi Obeagu.

**Supervision:** Emmanuel Ifeanyi Obeagu.

**Validation:** Emmanuel Ifeanyi Obeagu.

**Visualization:** Emmanuel Ifeanyi Obeagu.

**Writing—original draft:** Emmanuel Ifeanyi Obeagu.

**Writing—review & editing:** Emmanuel Ifeanyi Obeagu.
